
JOURNAL INFORMATION
==============================
NLM Title Abbreviation: Tex Med J (Austin)
NLM Journal ID: 21130590R

Texas Medical Journal

ISSN: 0892-8495
Publisher: Biomedical Journal Digitization Project

ARTICLE INFORMATION
==============================
PMCID: PMC9591405
Article version: 1
Subjects: Book Notices

Lessons in Practical Diagnosis

Print publication date: 1893 Aug
Volume: 9
Issue: 2
First page: 93
Last page: 93
License: This work is free of known copyright restrictions. For more information, please see PMC Back Issue Digitization.
License URL: https://creativecommons.org/publicdomain/mark/1.0/

==============================

H.

---

LESSONS IN PHYSICAL DIAGNOSIS. By Alfred L. Loomis, M. D., LL. D., Professor of the Practice of Medicine and Pathology in the University of the city of New York. Tenth edition, revised and enlarged. Octavo. Illustrations, some in color. 240 pages, extra muslin, price $3.00. New York. William Wood & Company.

Loomis' Physical Diagnosis has been a standard work for many years, and this, the tenth edition, is larger and better than any former edition. The chapters on the heart and the urine have been rewritten, and much that is valuable to the student has been added. The author has added a chapter on clinical microscopy, beautifully illustrated with colored plates, which increases the value of the work very much. This book is so well and so favorably known that praise of it would be superfluous. It is a necessity to all physicians and medical students. The paper and mechanical work are fully up to the standard, and deserve especial commendation. H.

---

### Publishers' Notes.

**Henry's Tri-Iodides** is approved by the most eminent clinicians, as a most reliable formula in gouty rheumatic and lithæmic conditions.

---

**We** request the attention of our readers to Messrs. John Wyeth & Bro.'s advertisement, in this issue, relating to their Efferves-
